# Neural correlates of deception in social contexts in normally developing children

**DOI:** 10.3389/fnhum.2013.00206

**Published:** 2013-05-17

**Authors:** Susumu Yokota, Yasuyuki Taki, Hiroshi Hashizume, Yuko Sassa, Benjamin Thyreau, Mari Tanaka, Ryuta Kawashima

**Affiliations:** ^1^Graduate School of Education, Tohoku UniversitySendai, Japan; ^2^Research Fellow of the Japan Society for the Promotion of ScienceTokyo, Japan; ^3^Division of Developmental Cognitive Neuroscience, IDAC, Tohoku UniversitySendai, Japan

**Keywords:** deception, fMRI, mentalizing, children, social context

## Abstract

Deception is related to the ability to inhibit prepotent responses and to engage in mental tasks such as anticipating responses and inferring what another person knows, especially in social contexts. However, the neural correlates of deception processing, which requires mentalizing, remain unclear. Using functional magnetic resonance imaging (fMRI), we examined the neural correlates of deception, including mentalization, in social contexts in normally developing children. Healthy right-handed children (aged 8–9 years) were scanned while performing interactive games involving deception. The games varied along two dimensions: the type of reply (deception and truth) and the type of context (social and less social). Participants were instructed to deceive a witch and to tell the truth to a girl. Under the social-context conditions, participants were asked to consider what they inferred about protagonists' preferences from their facial expressions when responding to questions. Under the less-social-context conditions, participants did not need to consider others' preferences. We found a significantly greater response in the right precuneus under the social-context than under less-social-context conditions. Additionally, we found marginally greater activation in the right inferior parietal lobule (IPL) under the deception than under the truth condition. These results suggest that deception in a social context requires not only inhibition of prepotent responses but also engagement in mentalizing processes. This study provides the first evidence of the neural correlates of the mentalizing processes involved in deception in normally developing children.

## Introduction

Deception is the process by which one individual deliberately attempts to convince another to accept as true what the first individual knows to be false (Spence et al., 2001). Deception itself is related to the cognitive process whereby a true response is inhibited and a deceptive one is produced (Abe et al., 2006). However, deception, especially in social contexts, also involves intent to instill false beliefs. The instillation of false beliefs requires that one track the false beliefs that are being engendered in the other person (Ship et al., 2008). Therefore, deception of another person not only requires the inhibition of some responses and the production of others; it also entails consideration of the knowledge available to the other person, appreciation of another's mental state, and the ability to predict the behavior of another individual based on her or his mental state (Yirmiya et al., 1996; Ship et al., 2008). To understand and manipulate other people's behavior in terms of their mental state are cognitive skills known as mentalizing (Frith et al., 1991; Frith and Frith, 1999). This ability is required for deceptions that occur in a social context.

The results of previous behavioral experimental research undertaken from a developmental perspective indicate that the ability to engage in deception in a social context develops later than do abilities related to inhibition and mentalizing. Ahern et al. (2011) revealed that normally developing children aged 3 years and 6 months could inhibit prepotent responses (factual statements) and offer a counterfactual response without any intent to deceive. Moreover, the ability to mentalize developed at around 4 years in normally developing children (Baron-Cohen, 1995; Wellman et al., 2001). In terms of deceptive behavior in social contexts, normally developing children could deceive another person at around 5 years of age (Sodian et al., 1991; Peskin, 1992). Additionally, several neurodevelopmental disorders, such as autism spectrum disorders (ASD), involve deficits in mentalizing abilities. Previous studies on children with ASD have reported robust results showing impairments in the ability to mentalize (Baron-Cohen et al., 1985; Leslie and Frith, 1988; Happé, 1994) and engage in deceptive behavior (Baron-Cohen, 1992; Sodian and Frith, 1992; Yirmiya et al., 1996; Li et al., 2010) in this population. However, little is known about the neural correlates of deception and mentalizing, even in normally developing children. Therefore, a neuroimaging study on normally developing children would help to clarify the neural abnormalities involved in neurodevelopmental disorders.

To the best of our knowledge, no neuroimaging studies have been conducted on deception in a social context. Although a number of studies on the neural correlates of deceptive behavior (Spence et al., 2001; Langleben et al., 2002, 2005; Lee et al., 2002; Mohamed et al., 2006; Gamer et al., 2007; Abe et al., 2008; Hakun et al., 2008; Baumgertner et al., 2009; Kozel et al., 2009) have been conducted, these focused on the processes associated with deception, and they targeted adults. These studies revealed that deceptive responses were associated with increased activation in prefrontal regions and the anterior cingulate cortex (Spence et al., 2001; Langleben et al., 2002; Nunez et al., 2005; Abe et al., 2006; Ganis et al., 2008). Abe et al. (2006) suggested that the dorsolateral, ventrolateral, and medial prefrontal cortices; anterior cingulate cortex; and inferior parietal lobule (IPL) were related to response inhibition and production of deceptive replies. However, the neural correlates of deception in social contexts have not been well-studied, especially in children.

With respect to the processes associated with mentalizing, a number of functional brain studies have reported that mentalizing involves an extended network located bilaterally in the frontal, temporal, and parietal lobes. Previous studies reported activations which reflected that in the temporoparietal junction (TPJ) was associated with reading intentions from action (Lissek et al., 2008; Bahnemann et al., 2009), the superior temporal sulcus (STS) was related to the processing of visual-spatial information (Pelphrey et al., 2004; Bahnemann et al., 2009), and the precuneus was related to adopting the perspective of another person (Cavanna and Trimble, 2006). The region from the medial prefrontal and anterior cingulate cortices to the paracingulate cortex has been associated with making inferences about another's thoughts (Fletcher et al., 1995; Amodio and Frith, 2006).

Despite previous neuroimaging research that has clarified the neural correlates of deception and mentalizing in adults, questions about whether the neural correlates of deception and mentalizing in normally developing children rely on same brain regions as they do in adults remain unanswered. However, no functional magnetic resonance imaging (fMRI) study has yet focused on deception in normally developing children in social contexts. Thus, the aim of this study was to investigate whether the neural correlates of deception and mentalizing in social contexts were the same in normally developing children as in adults. We also examined whether the neural correlates of mentalizing were modulated by reply type (deceptive or truthful statements).

To these ends, we conducted a rapid event-related fMRI experiment using an interactive game that involved a set of animated stimuli associated with deception. Two factors were studied: type of reply (deception or truth) and type of context (social or less social). We tested the following three hypotheses: (1) Activations in the dorsolateral, ventrolateral, and medial prefrontal cortices; anterior cingulate cortex; and IPL would be associated with the main effect of deception. These activations would reflect inhibition of prepotent response processing. (2) Activations in the medial prefrontal cortex, STS, TPJ, temporal pole, and precuneus would be associated with the main effect of social context and would be related to perspective taking, inference, and anticipation of the mental state of another. (3) The brain regions underpinning both deception and mentalizing would integrate the cognitive operations required for deception in a social context. Ramnani and Owen (2004) concluded that the medial aspect of the prefrontal cortex plays a key role in integrating the outcomes of two or more separate cognitive operations in the pursuit of a higher-level behavioral goal. Therefore, we assumed that the medial aspect of the prefrontal cortex would be responsible for integrating the processing involved in deception and mentalizing and that the development of this region would be related to the ability to engage in deception in social contexts.

## Methods

### Participants

The sample consisted of 28 healthy children aged 8 or 9 years (nine females; mean age = 8.9, *SD* = 0.59; mean IQ = 106.46, *SD* = 10.29) recruited with local advertisements. All had normal vision, and none had a history of neurological or psychiatric illness. All participants were right-handed according to the Edinburgh Handedness Inventory (Oldfield, 1971). Their IQ was assessed by the WISC-III. Data from six participants were discarded due to excessive head motion (>3 mm), and data from 12 other participants were discarded because of poor performance on tasks (<70% accuracy; see Behavioral Results section). The final sample therefore comprised 10 participants. Mean age was 113.4 months (*SD* = 12.5, range = 97–128). There was no group difference between the final and discarded participants [*M* = 111.3, *SD* = 11.5; *t*_(26)_ = 0.44, *P* = 0.66]. Additionally there was no group difference in relation to full scale IQ [final participants: *M* = 106.3, *SD* = 9.5, discarded sample: *M* = 106.9, *SD* = 10.9; *t*_(26)_ = 0.06, *P* = 0.95]. All children were paid for their participation. Each participant and his/her parent signed an informed consent form. This study was approved by the Ethics Committee of Tohoku University.

### Materials

**Figure 1:**
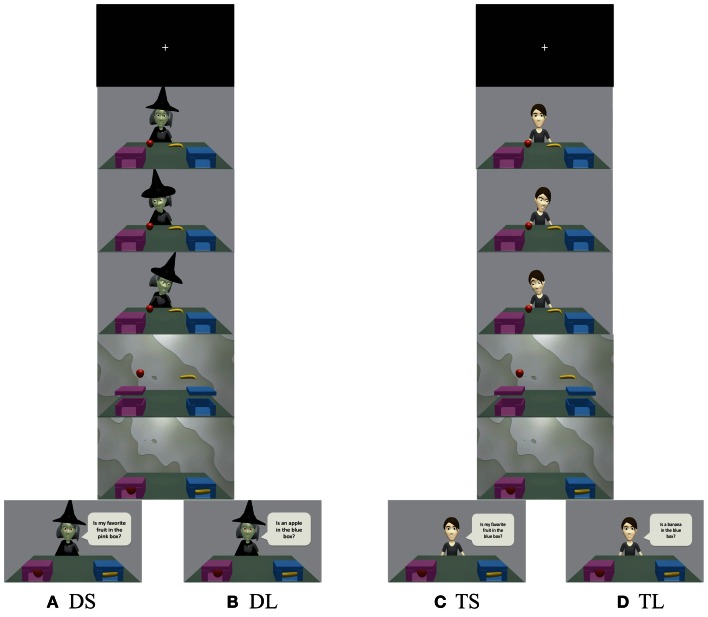
**Time courses for (A) deception with social-context (DS), (B) deception with less-social-context (DL), (C) truth with social-context (TS), and (D) truth with less-social-context (TL).** Participants were instructed to respond truthfully to the girl's questions and to deceive the witch. Under the social conditions (DS and TS), protagonists asked about the location of their liked or disliked fruit. On the other hand, under the less-social conditions (DL and TL), protagonists asked about the location of a certain fruit. See the Materials and Methods section for additional details.

We modified the behavioral experiment conducted by Sodian and Frith (1992) and used two boxes for the animation (Figure 1). The protagonists in the animation were a witch and a girl, and we used blue and pink boxes featuring a window facing participants to decrease memory load. Two kinds of fruit (chosen randomly from apples, bananas, and oranges) appeared on a table. In the first scene of the animation, a protagonist sat in front of the table, on which the boxes and fruit were placed. Next, the protagonist looked at one fruit with a smile or an expression reflecting disgust. She then looked at the other fruit with a facial expression opposite the first (if she smiled at the first fruit, she expressed disgust toward the second). These procedures allowed participants to judge which fruit the protagonist liked and which she disliked. Next, the fruit “jumped” into the boxes after a curtain was drawn. Because the protagonists were behind the curtain, they were unable to see which box contained which fruit. However, participants were able to view this sequence of events. In the last scene, the curtain opened and the protagonist asked participants which box contained which fruit. Under the deception with social-context (DS) and truth with social-context (TS) conditions, protagonists asked participants about the position of their favorite fruit: “Is my favorite fruit in the pink box?” Under these conditions, participants should have been able to infer the protagonist's preference from his or her facial expression and use this information to answer the question correctly. On the other hand, under the deception with less-social-context (DL) and truth with less-social-context (TL) conditions, protagonists simply asked about the position of a certain fruit: “Is the apple in the blue box?” Under these conditions, participants did not need to consider the protagonists' preference.

We adopted a rapid event-related design for fMRI data collection. One trial took 13 s (animation: 8 s, response time: 5 s). Trials were presented in a pseudo-randomized order with a jittered inter-stimulus interval (ISI, minimum 2 s, maximum 6 s, mean 4 s). Sixteen trials were conducted under each condition, yielding a total of 64 trials (16 trials × 4 conditions).

### Procedure

Participants were asked to watch the short animated film and answer a simple yes/no question for each trial. The girl was introduced as a good character who should be helped to get the fruit she sought. The witch was introduced as an evil character who should be prevented from getting the fruit. Participants were told, “In the animation, there were two protagonists: a witch and a girl. They looked for some fruit, but they did not know where it was. So, if the girl asks you about the position of the fruit, you should always tell it to her. But whenever the witch asks you about the position of the fruit, you should prevent from her getting it.” Before participants entered the scanner, they completed a short practice task consisting of two trials under each condition; the trials were self-paced to maximize task comprehension. They also completed two additional trials per condition to practice answering within 5 s. If they failed to answer the question correctly, we presented the rules and explained the situation again. We confirmed that all participants could answer all practice questions correctly. To decrease fatigue, we divided the fMRI session into two sessions. Half of the participants were pseudo-randomly assigned to the DL/TL condition in the first session, and the other half were pseudo-randomly assigned to the DS/TS condition in the first session. Additionally, to reduce nervousness or anxiety during scanning, participants looked at the MRI scanner and listened to its scanning sound before entering the MRI machine.

The animations were presented visually using customized experimental control software (Presentation, Neurobehavioral Systems, Inc., Albany, CA, USA). Data on the accuracy and response time for all tasks were collected by this software.

### fMRI data acquisition

All MRI data were acquired with a 3-T Philips Intera Achieva scanner. Head motion was minimized by placing pillows and cushions around the head. Forty axial slices (thickness, 4 mm; FOV, 224 mm; data matrix, 64 × 64 voxels; in-plane resolution, 3.5 × 3.5 mm) were acquired every 2.2 s during functional measurements (single-shot gradient-echo echoplanar imaging sequence; *TR* = 2200 ms; *TE* = 30 ms; flip angle = 80°). Within one session, 225 volumes were acquired.

### fMRI analyses

The data were analyzed using Statistical Parametric Mapping Software version 8 (Wellcome Department of Cognitive Neurology, London, UK) implemented on MATLAB (The Mathworks, Inc., Natic, MA, USA). Acquired volumes were subjected to a standard preprocessing procedure (Ashburner and Friston, 1997). Images were corrected for differences in timing of slice acquisition, followed by rigid body motion correction. Preprocessing further included spatially normalization to the standard Montreal Neurological Institute (MNI) adult EPI template and smoothing with an isotropic Gaussian kernel of 8 mm (FWHM). The reason why we used adult template for normalization was that transformed brain morphology into a common stereotactic space is relatively consistent between children and adults (Burgund et al., 2002). Therefore, we used the standard MNI EPI template for normalizing our data.

fMRI data ware analyzed using an event-related model. In the first-level analysis, we created and estimated a general linear model (GLM). In the creation of the GLM, the hemodynamic response for each event was modeled from the onset of the appearance of the question to the minimum response time under all four conditions. Participants were given 5 s to press the response button. If participants could answer the question within 3 s, they no longer needed to consider the question. We used this minimum response time to eliminate cognitive processing following answers. Trials with errors or no response were modeled as events of no interest. A high-pass filter of 1/128 Hz was used to remove low-frequency noise. In the first-level analysis, we created the contrast between brain activations under DS + DL and TS + TL conditions for each participant in order to identify the main effect of deception. Additionally, to identify the main effect of social context, we compared the contrast under DS + TS and DL + TL conditions. In the interaction comparisons, the contrast (DS − DL) − (TS − TL) was used. We also created following contrasts; DS − DL, DS − TS, DS − TL, and TS − TL to use for mask images.

To assess the neural activities under deception and social-context conditions, we performed second-level one-sample *t*-test analyses by using the first-level contrasts. First, the main effect of deception was determined by comparing brain activity under the deception (DS + DL) and the truth (TS + TL). This contrast was masked inclusively by DS − TS and DL − TL for the main effect of deception. In a similar way, to investigate the neural correlates of social context, we compared the social (DS + TS) and the less-social (DL + TL). This contrast was masked inclusively by DS − DL and TS − TL. Second, in the interaction effect between deception and social context was determined using the contrast (DS − DL) − (TS − TL) with inclusive mask of DS − DL, DS − TS, and DS − TL. This contrast and mask procedure enabled us to identify brain areas that showed greater difference among DS and other three conditions.

For all the whole-brain subtraction analyses, the statistical threshold was set at *P* < 0.001 for height, and the FWE was corrected to *P* < 0.05 for multiple comparisons using cluster size, assuming the entire brain as a search volume. A liberal statistical threshold for the mask contrasts was set at *P* < 0.05 for height, without a correction for multiple comparisons.

## Results

### Behavioral data

We performed a Two-Way ANOVA with reply type (deception/truth) and context (social/less social) as the independent variables and accuracy as the dependent variable. We found a significant main effect of context [*F*_(3, 36)_ = 13.18, *P* < 0.001; Table 1]. Participants performed significantly better under the less-social than under the social conditions. We also performed a Two-Way ANOVA with reply type and context as independent variables and reaction time as the dependent variable. The analysis revealed a significant main effect of reply type and context [reply type: *F*_(3, 36)_ = 5.44, *P* < 0.05; context: *F*_(3, 36)_ = 25.48, *P* < 0.001; Table 1]. Participants responded to the question faster under the truth and less-social conditions than under the deception and social conditions.

**Table 1:** **Behavioral data**.

**Variable**	**Deception**		**Truth**	
	**Social**	**Less-social**	**Social**	**Less-social**
Accuracy (trials)	13.5 ± 1.51	15.7 ± 0.48	14.3 ± 0.82	15.7 ± 0.48
Reaction time (s)	4.09 ± 0.34	3.45 ± 0.54	3.82 ± 0.33	3.1 ± 0.46

Mean accuracy score and reaction time under each condition are shown. Values are means ± standard deviations.

### fMRI data

**Figure 2:**
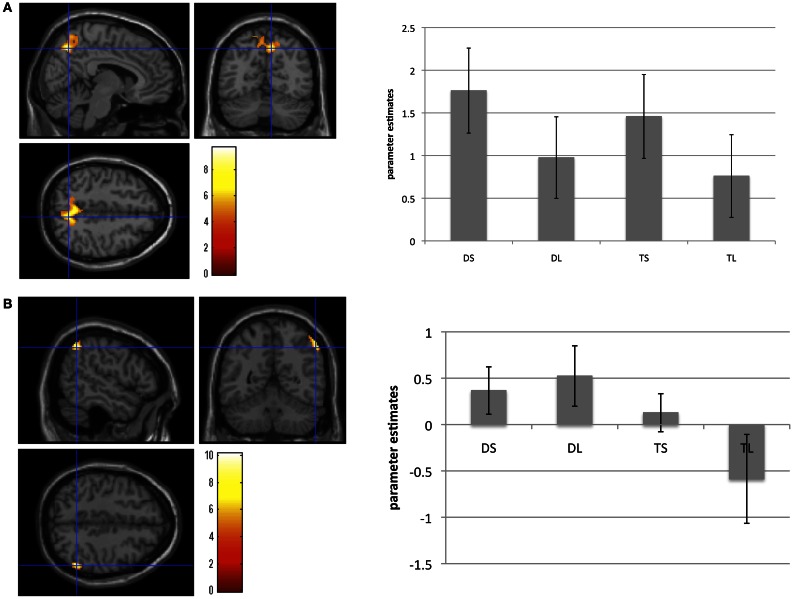
**Brain areas showing brain activation during (A) the social conditions compared with the less-social conditions, and (B) the deception conditions compared with the truth conditions.** Activation in the right precuneus areas was significantly greater under the social than under the less-social conditions. Activation in the right inferior parietal lobule was marginally greater under the deception than under the truth conditions. These bar graphs showed the parameter estimates of each condition in the right precuneus and the right inferior parietal lobule. Error bars meant standard errors.

The second-level group analysis revealed significant activation for the main effect of mentalizing. We found significant right precuneus activation for the (DS + TS) – (DL + TL) contrast. We found significant IPL activation for the (DS + DL) – (TS + TL) contrast using the more liberal statistical threshold (*P* < 0.001, FWE corrected *P* < 0.1 by cluster size). However, we did not find any significant interactions. Brain areas showing significant or marginally significant activation are listed in Table 2 and Figure 2.

**Table 2:** **Cortical areas activated as the main effect under social and deception conditions**.

**Contrast/area**	**Peak activation**				**Cluster size (mm^3^**)
	**Coordinates**			***t***	
	***x***	***y***	***z***		
**SOCIAL (DS AND TS) – LESS-SOCIAL (DL AND TL)**					
R precuneus^*^	8	−64	48	9.70	1112
	2	−56	48	8.72	
	24	−58	30	6.76	
**DECEPTION (DS AND DL) – TRUTH (TS AND TL)**					
R Inferior parietal lobule^†^	54	−54	46	10.14	156
	50	−56	56	7.39	

DS, deception with social context; TS, truth with social context; DL, deception with less-social-context; TL, truth with less-social-context.

* P < 0.001 corrected by cluster level P < 0.05.

† P < 0.001 corrected by cluster level P < 0.1.

## Discussion

To the best of our knowledge, this is the first study investigating the neural correlates of deceptive behavior in social contexts in normally developing children. Overall, the results were partially consistent with the hypotheses. First, the comparison between social-context conditions and less-social-context conditions showed that the right precuneus was significantly more activated under the social than under the less-social condition. Second, we found marginally greater IPL activation under the deception compared with under the truth conditions. Since the interaction between deception and social context did not reach a significant level, the hypothesis that the medial aspect of the prefrontal cortex was responsible for integrating the process involved in deception and mentalizing was not proven in normally developing children.

### Precuneus activation in the social context conditions

The activation of the precuneus observed under the social-context conditions suggested that this region is related to the cognitive processes underlying taking the perspective of another person. Indeed, this region has been among the areas associated with mentalizing (Saxe, 2006; Frith and Frith, 2007). Participants in the present study were required to infer the preference of the protagonist from the character's facial expression. Additionally, some researchers have deduced that the precuneus plays a role in the cognitive processing involved in retrieving past events, especially those experienced by others (Shallice et al., 1994; Lundstrom et al., 2005). These cognitive processes would be required to respond to the question asking participants about the preference of the protagonist. On the other hand, Mohamed et al. (2006) speculated that precuneus activation was related to the ability to determine whether one's own mental imagery is correct. They found greater activation in the precuneus when they contrasted reactions to a lie about a mock crime (e.g., firing a gun) with those to the truth. In that study, participants retrieved information about their own behavior to offer a deceptive reply about whether they had actually committed a mock crime. However, in the present study, the tasks involved cognitive processing about protagonists' rather than participants' mental imagery. Therefore, it is less likely that precuneus activation observed in the present study was involved in the mental imagery related to the participants themselves. In this context, we assumed that the observed precuneus activation reflected the processes involved in taking the perspective of another person and in retrieving the information needed to infer the preference of the protagonist.

### IPL activation in the deception conditions

We found that activation in the IPL of normally developing children was associated with processing related to deception. This result is partially consistent with previous fMRI studies in adults that found IPL activations were associated with deceptive behavior (Spence et al., 2001; Lee et al., 2002; Kozel et al., 2005; Nunez et al., 2005; Abe et al., 2006) and with executive functioning, especially when response inhibition was involved (Rubia et al., 2001; Simmonds et al., 2008). These studies assumed that IPL activation was responsible for the inhibition of the prepotent response. Simmonds et al. (2008) hypothesized that frontal–parietal circuits were recruited in response to inhibition tasks, especially when working memory was necessary to guide response inhibition. Consistent with these results, the present study found that IPL activation indicated inhibition of a prepotent (true) response to deceive another person.

Contrary to our hypotheses, we did not find a main effect of activation in the prefrontal regions associated with deception, the social context, or the interaction of deception and social context. This may have been due to the immaturity of prefrontal regions. A number of investigators have examined the differential maturation of response inhibition in frontal and parietal cortices (Booth et al., 2003; Rubia et al., 2006). Bunge et al. (2002) compared the brain activation exhibited by 8–12-year-old children during an inhibition task with that exhibited by adults and concluded that adults activated frontal and parietal regions during an inhibition task, whereas children activated only parietal regions. Additionally, only a few studies have investigated differences between adolescents and adults in the brain correlates of mentalizing (Ohnishi et al., 2004; Wang et al., 2006; Williams, 2008; Sommer et al., 2010; van den Bos et al., 2011; Moor et al., 2012; Sebastian et al., 2012). These studies indicated that young participants (aged 10–12 years) tended to show more activation in prefrontal regions than did adults (Sommer et al., 2010; Moor et al., 2012) and that the activation pattern shifted from prefrontal to occipitotemporal regions during development (Wang et al., 2006; Sebastian et al., 2012). Those researchers assumed that this shift occurred due to the increasingly automatic nature of the cognitive processing. In the present study, we did not find any significant activation in the prefrontal regions. However, the participants in previous studies were older than 10 years of age and thus older than participants in the present study. Taki et al. (2011) found that prefrontal regions develop later than do other brain areas. Taken together, the results of the present study may be interpreted as indicating that children younger than 10 years do not use the prefrontal regions efficiently because of the immaturity of these regions. As the prefrontal regions mature, activations in these regions would be expected to increase. Subsequently, increases in automatic cognitive processing would be associated with decreased activation in the prefrontal regions.

### Limitations

One limitation of the present study is its small sample size for statistical analyses. Therefore, a larger sample study is needed in order to confirm these findings in future studies. We eliminated 12 participants (43%) due to problems with accuracy. This ratio is relatively larger than that in other studies of children (Sommer et al., 2010; Sebastian et al., 2012). We assumed that the unsuccessful trials of some participants may have been due to the unfamiliarity of the MRI environment (e.g., sound of the scanner, darkness, or narrow space) rather than to task difficulty. Participants practiced with same situation used for fMRI tasks (i.e., they watched the same animated video and used the same response pad) for four trials per condition (total of 16 trials). During the practice phase, we confirmed that participants could answer the question with 100% accuracy compared with only 70% accuracy during scanning. Therefore, we concluded that participants found it difficult to concentrate on the tasks due to the MRI environment. In order to get use to scanning environment, the training in a mock scanner might be required for young children.

Secondly there might be a potential confond between conditions and the character in our tasks. Because the girl always appeared in the truth conditions (TL and TS) and the witch always appeared in the deception conditions (DL and DS), the processing involved in seeing a novel face did not cancel out in the subtraction of deception minus truth; (DS + DL) – (TS + TL). However, the neural activities such as novel face processing (e.g., witch's face in the present study) were found in right hippocampus, left prefrontal and temporal cortices (Grady et al., 1995), right hippocampus and bilateral prefrontal cortices (Haxby et al., 1996). Therefore, we considered that the activation in right IPL that we found as deception processing in the present study might be related to the processing of deception itself, not novel face.

## Conclusion

To the best of our knowledge, this is the first study to clarify the neural correlates of deception in social contexts in normally developing children. We found that processing in social contexts involves the precuneus. Taken together with the results of previous fMRI studies on mentalizing, our results suggest that this region played a role in the perspective taking and memory retrieval required to infer the protagonist's preference. Additionally, we found marginal activation in the right IPL during deception processing, which we related to the inhibition of prepotent responses. These results contribute to clarifying an essential aspect of deceptive behavior in normally developing children. Moreover, these results should be helpful in investigations of neural abnormalities in children with neurodevelopmental disorders such ASD. Contrary to our hypothesis, we did not find any significant activation of interaction between deception and social contexts. This might suggest that deception is not modulated by social factors.

### Conflict of interest statement

The authors declare that the research was conducted in the absence of any commercial or financial relationships that could be construed as a potential conflict of interest.
